# Comprehensive Characterization and Antioxidant Function Prediction of Endogenous and Exogenous Peptides from *Polygonatum kingianum*

**DOI:** 10.3390/cimb48070735

**Published:** 2026-07-19

**Authors:** Jieyao Ma, Huiling Liu, Yalan Wu, Tingsheng Ma, Huaming Xiao, Wei Cai

**Affiliations:** 1 School of Pharmaceutical Sciences, Hunan University of Medicine, Huaihua 418000, China; majieyao@hnmu.edu.cn (J.M.); 19372318251@163.com (H.L.); wuyalan5@163.com (Y.W.); matingsheng1111@163.com (T.M.); 2 School of Bioengineering and Health, Wuhan Textile University, Wuhan 430200, China

**Keywords:** *Polygonatum kingianum*, peptides, nano-liquid chromatography–high resolution mass spectrometry, bioinformatics analysis, molecular docking

## Abstract

**Background/Objectives:** *Polygonatum kingianum* Collett & Hemsl. (PK) is an edible medicinal herb with tonic effects. Its natural antioxidant peptides are valuable for functional food development, while their characteristics and mechanisms are unclear. This study aimed to identify PK antioxidant peptides and explore their antioxidant molecular mechanisms to support the utilization of PK active ingredients. **Methods:** Three peptide fractions (P1, P2, P3) were prepared from PK via defatting, alkali–acid precipitation and enzymatic hydrolysis. Nano-liquid chromatography coupled with Q Exactive mass spectrometry was used for peptide identification. Bioinformatic tools predicted peptide antioxidant activity, and molecular docking targeting the Kelch-like ECH-associated protein 1-nuclear factor erythroid 2-related factor 2 (Keap1-Nrf2) pathway verified peptide–target-binding affinity. **Results:** A total of 747, 1850 and 2537 peptides were identified from P1, P2 and P3, respectively, among which 119 peptides were screened out as potential antioxidant candidates. Docking analysis revealed 10 peptides with strong binding affinity to Keap1. These active peptides were short sequences of 3–5 residues enriched in hydrophobic and aromatic amino acids, which stably bound key residues within the Keap1 binding pocket. **Conclusions:** This study fills the research gap in PK peptidome profiling, clarifies structural signatures of candidate antioxidant peptides, and identifies high-affinity short peptides targeting the Keap1-Nrf2 antioxidant pathway. The established screening pipeline provides technical support for bioactive peptide mining and deep processing of PK resources.

## 1. Introduction

*Polygonatum kingianum* Collett & Hemsl. (PK), a member of the Liliaceae genus, *Polygonatum*, is well-documented in traditional Chinese medicine (TCM) for its functions of tonifying qi, nourishing yin, moistening the lungs, and tonifying the kidneys. As a characteristic medicinal plant native to Southwest China, PK possesses high resource and economic value. Modern pharmacological studies confirm that PK exerts multiple bioactivities, including immunomodulation, anti-inflammation, hypoglycemia, lipid-lowering and anti-aging effects, among which antioxidant capacity serves as the core basis of its pharmacological actions [1]. PK extracts exhibit strong antioxidant capacity (12.6 mmol Fe^2+^/g), upregulate antioxidant enzymes (superoxide dismutase [SOD], catalase [CAT], glutathione peroxidase [GSH-Px]), reduce accumulation of reactive oxygen species (ROS) and malondialdehyde (MDA), and alleviate oxidative hepatocyte damage [2]. In vivo assays demonstrate crude PK extracts mitigate oxidative stress in hyperlipidemic mice and hypoxic rats [3]. Polysaccharides are the primary bioactive components of PK, whose molecular structures and glycan conformations have been partially characterized [4]. PK also contains characteristic steroidal saponins such as dioscin and methyl protodioscin [5]. Li et al. identified 153 PK chemical constituents via high-resolution mass spectrometry, including saponins, flavonoids, phenolic acids and 37 peptides [6]. Recent studies on *Polygonatum* peptides have expanded rapidly; abundant endogenous peptides have been detected in *P. cyrtonema* and *P. odoratum*, with short oligopeptides of 4–6 amino acids as the dominant bioactive components [7,8]. Nevertheless, research on PK peptides lags far behind that of congeneric species, with two major research gaps remaining. First, no high-coverage species-specific PK peptide dataset has been constructed, hindering novel bioactive peptide screening. Second, while the antioxidant effects of PK polysaccharides, saponins and flavonoids have been validated, the antioxidant activity and underlying mechanisms of PK peptides remain unelucidated. Therefore, systematic identification and antioxidant function prediction of endogenous and exogenous PK peptides are urgently needed.

Bioactive peptides are low-molecular compounds consisting of 2–50 amino acid residues linked by peptide bonds, widely distributed in plant tissues. They are categorized into endogenous and exogenous peptides based on biosynthetic origins [9,10]. Endogenous peptides are synthesized during plant growth and development, mediating physiological processes including biotic/abiotic stress defense and signal transduction [11,12]. Exogenous peptides are mainly generated from plant proteins during processing, such as enzymatic hydrolysis and fermentation, or via chemical/recombinant synthesis. These peptides exhibit excellent intestinal mucosal permeability, rapid human absorption, high bioavailability and favorable safety profiles [13,14,15]. Compared with synthetic peptides, plant-derived natural bioactive peptides show superior structural stability, lower toxicity, better metabolic safety and optimized absorption performance. Most importantly, they exert multi-target antioxidant activity, alongside antihypertensive, lipid-lowering, antibacterial, gut microbiota-regulating and immunomodulatory effects via regulating in vivo metabolic and signaling pathways [16]. Systematic identification and functional characterization of PK antioxidant peptides can expand the library of natural redox regulators, clarify the bioactive mechanisms of *Polygonatum* herbs, and facilitate the development of PK-derived nutraceuticals and redox-targeted therapeutic agents.

Mass spectrometry (MS) is the mainstream analytical platform for bioactive peptide identification, featuring high sensitivity and resolution to detect trace peptides in complex samples and provide accurate sequence information for bioactive screening [17,18,19]. MS spectra are commonly analyzed via database searching, which matches experimental fragment ion signals with reference peptide sequences to enable rapid, high-precision identification [20]. De novo sequencing eliminates database dependence by reconstructing peptide sequences directly from tandem MS (MS/MS) fragment patterns. This method identifies unannotated peptide sequences absent from existing protein databases, expands the range of detectable peptides, overcomes incomplete genomic/transcriptomic annotation limitations, and improves the discovery efficiency of novel uncharacterized bioactive peptides [21]. Combining MS identification with computational tools improves antioxidant peptide screening efficiency and reliability. AnOxPePred 1.0 is a dedicated deep learning tool for predicting peptide free radical-scavenging capacity with clear functional screening criteria [22]. PeptideRanker adopts convolutional neural networks to evaluate overall peptide bioactivity, excluding peptides with strong radical-scavenging capacity but negligible in vivo physiological effects [23,24]. Combined use of the two tools realizes dual screening of “functional specificity + valid bioactivity” to establish an accurate preliminary screening system. Molecular docking further reveals atomic-level peptide–target interaction mechanisms [25]. The Keap1-Nrf2 pathway is the core cellular antioxidant signaling axis: under physiological conditions, Keap1 suppresses Nrf2 activity; disrupting their interaction activates downstream antioxidant gene expression [26]. Docking against Keap1 (the primary negative regulator of Nrf2) provides structural evidence for peptide antioxidant activity and guides subsequent functional verification.

Based on previous research on *Polygonatum* antioxidant peptides [27], this study established an integrated workflow combining high-resolution MS, multi-layer bioinformatic analysis and molecular docking to systematically screen PK antioxidant peptides. Three pretreatment strategies were developed to enrich free endogenous peptides, matrix-bound peptides and enzymatically released exogenous peptides. Nano-liquid chromatography coupled with Q-Orbitrap MS combined database searching and de novo sequencing to maximize peptide identification coverage. A hierarchical screening pipeline integrating AnOxPePred and PeptideRanker was applied to select candidate antioxidant peptides, followed by Keap1-targeted molecular docking to quantify binding affinity and characterize sequence–activity relationships between peptide structural features and Keap1 binding. This work constructs a targeted technical system for PK resource utilization and a high-throughput screening platform for natural antioxidant peptides, providing core data for subsequent functional validation and deep excavation of PK bioactive peptides.

## 2. Materials and Methods

### 2.1. Chemical Reagents

PK was collected from Huaihua, Hunan Province, China, and subsequently authenticated by Dr. Wei Cai of Hunan University of Medicine. Petroleum ether was purchased from Shanghai Aladdin Biochemical Technology Co., Ltd. (Shanghai, China). Sodium hydroxide was purchased from Sinopharm Chemical Reagent Co., Ltd. (Shanghai, China). Hydrochloric acid was purchased from Shanghai Lingfeng Chemical Reagent Co., Ltd. (Shanghai, China). HPLC-grade acetonitrile was purchased from Thermo Fisher Scientific (Shanghai, China) Co., Ltd. LC-MS-grade formic acid was purchased from Thermo Fisher Scientific (Shanghai, China) Co., Ltd. HPLC-grade trifluoroacetic acid was purchased from TCI Development Co., Ltd. (Shanghai, China). C18 microcolumns (ZTC18S096) were purchased from Merck Millipore (Shanghai, China) Co., Ltd. Ultrapure water was prepared using a Milli-Q Integral 10 ultrapure water system (Merck Millipore, Darmstadt, Germany).

### 2.2. Sample Preparation

Dried PK was pulverized using a grinder (FSJ-1000C, Lingsum, Yongkang Hongtaiyang Electromechanical Co., Ltd., Yongkang, China) and sieved through 0.178 mm. The collected powder was defatted with petroleum ether at a solid-to-liquid ratio (*w*/*v*) of 1:3 for three cycles (5 min each) with continuous stirring until the petroleum ether turned clear. After filtration (SHZ-III, Jinye, Shanghai Yarong Biochemical Instrument Factory, Shanghai, China), the defatted PK powder was air-dried to obtain the defatted PK sample (P1).

A total of 10 g of the defatted powder was mixed with ultrapure water at a solid-to-liquid ratio (*w*/*v*) of 1:22 and incubated at 40 °C. The extraction was performed for 40 min with assisted ultrasonication (DZKW-S-Z, Yongguangming, Beijing Ever Bright Medical Treatment Instrument Co., Ltd., Beijing, China) at 100 W, and the pH was adjusted to 11 with 1 M sodium hydroxide solution. The extract was centrifuged at 4000 rpm for 25 min using a centrifuge (L530 R, Cence, Hunan Xiangyi Experimental Instrument Development Co., Ltd., Changsha, China), and the supernatant was collected. The pH of the supernatant was adjusted to 3 with 1 M hydrochloric acid, followed by standing for 1 h to precipitate crude protein. After centrifugation at 4000 rpm for 10 min, the supernatant was collected and freeze-dried (LGJ-10C, Foring, Foring Technology Development (Beijing) Co., Ltd., Beijing, China) into powder to obtain the PK sample (P2). Meanwhile, the precipitate was collected and reserved for the subsequent preparation of sample P3.

The PK sample (P2) was subjected to enzymatic hydrolysis following our previous method [27]: alkaline protease was added at 4500 U/g, the liquid-to-solid ratio was 75 mL/g, the hydrolysis pH was 8.4, and incubation was carried out at 37 °C for 4 h. After hydrolysis, the mixture was centrifuged at 4000 rpm for 20 min, and the supernatant was collected and freeze-dried to obtain the PK enzymatic hydrolysate sample (P3).

All PK rhizome raw materials were collected from multiple sites in Huaihua and fully homogenized before pulverization to eliminate individual biological differences in single plants. One batch of powder was prepared for each fraction for subsequent desalination and LC-MS analysis.

### 2.3. Sample Desalination

50 mg of each sample (P1, P2, P3) was dissolved in 200 μL of 0.1% trifluoroacetic acid (TFA) solution. Desalination was performed using a C18 microcolumn: the column was rinsed 10 times sequentially with 50 μL of 60% acetonitrile containing 0.1% TFA and 10 μL of 0.1% TFA. After the sample solution was aspirated and released repeatedly 20 times, the column was rinsed 5 times with 10 μL of 0.1% TFA. Peptides were eluted with 10 μL of 60% acetonitrile containing 0.1% TFA, followed by freeze-drying.

The lyophilized powder was reconstituted in 20 μL of 5% acetonitrile containing 0.1% TFA, vortex-mixed (VORTEX-5, Kylin-Bell, Haimen Kylin-Bell Lab Instruments Co., Ltd., Haimen, China), and centrifuged at 13,500 r·min^−1^ for 20 min at 4 °C (5424R, Eppendorf, Eppendorf (Shanghai) International Trade Co., Ltd., Shanghai, China). The supernatant was collected for subsequent LC-MS/MS analysis.

### 2.4. Nano-Liquid Chromatography–Quadrupole–Orbitrap Mass Spectrometry Analysis

Peptides in PK powder and crude protein powder were analyzed using a Q-Exactive Plus Quadrupole–Orbitrap MS (Thermo Fisher Scientific, Bremen, Germany). The system was coupled online with an EASY-nLC 1200 system (LC140, Thermo Scientific, Bremen, Germany) equipped with a home-made C18 column (75 μm i.d., 150 mm length) packed with Acclaim PepMap RSLC C18 (2 μm particle size, 100 Å pore size, Nanoviper). LC-MS conditions were set as follows: mobile phase A—aqueous 0.1% formic acid; mobile phase B—80% acetonitrile containing 0.1% formic acid. The flow rate was maintained at 400 μL/min with gradient elution: 0–3 min, 3% B; 3–7 min, 3–8% B; 7–46 min, 8–32% B; 46–51 min, 32–44% B; 51–60 min, 44–99% B; and 60–65 min, 99–3% B. Injection volume: 8 μL. Each sample was detected once by on-board injection. MS data acquisition parameters: Full MS scans were recorded from *m*/*z* 350 to 1550 at a resolution of 120,000. The top 20 most abundant precursor ions were fragmented in data-dependent acquisition (DDA) mode. Tandem MS resolution was set to 30,000, with a normalized collision energy (NCE) of 32%.

### 2.5. Data Analysis

Raw MS files were imported into PEAKS Studio 11 (version 11.5), and peptide identification was performed using both database search and de novo sequencing methods. For database search, the UniProt database with taxonomy ID 16195 was used, with a precursor ion mass tolerance of 15 ppm and a fragment ion mass tolerance of 0.05 Da; no protease cleavage site was specified. The “Deep Learning Enhancement” option was enabled during database search to improve the efficiency of peptide identification and the accuracy of peptide–spectrum matches (PSMs) using an enhanced deep learning algorithm. For database search identification results, peptides were filtered with the cut-off values of −10lgP ≥ 20 and peak area of ≥ 10^4^. Notably, no extra global 1% FDR filter was used post database searching. The −10lgP metric is PEAKS Studio’s native confidence score calculated as −10log_10_(P) (P = false matching probability per PSM). Setting −10lgP ≥ 20 limits single PSM false matches to *p* ≤ 0.01, achieving equivalent 1% error control to standard 1% FDR [28,29,30]. For de novo sequencing identification results, the confidence thresholds were set as average local confidence (ALC) ≥ 70% and single amino acid confidence threshold ≥60%. Additionally, a fault-tolerant strategy was adopted that only a maximum of one low-to-medium confidence residue with a confidence value ranging from 60% to 70% was retained for each de novo sequenced peptide.

### 2.6. Bioinformatics and Peptide Functional Prediction Analysis

The online tool IPC 2.0 (https://isoelectric.org/index.html, accessed on 12 July 2025) was used to calculate the isoelectric point and charge of peptides. The grand average of hydropathy (GRAVY) value was predicted via the GRAVY Calculator (https://www.gravy-calculator.de/index.php, accessed on 12 July 2025). AnOxPePred 1.0 (https://services.healthtech.dtu.dk/services/AnOxPePred-1.0, accessed on 18 July 2025) was employed to predict the antioxidant potential of peptides based on parameters including a free radical scavenging rate greater than 0.5, with prediction and scoring implemented using a convolutional neural network approach. PeptideRanker was subsequently used to predict general bioactivity probability (https://distilldeep.ucd.ie/PeptideRanker, accessed on 18 July 2025), and peptides with a score exceeding 0.7 were screened for sequence difference analysis.

### 2.7. Molecular Docking Simulation

The three-dimensional crystal structure of Keap1 Kelch-Neh2 complex (PDB ID: 2FLU) was downloaded from the Protein Data Bank (https://www.rcsb.org/, accessed on 25 July 2025). PyMOL (v1.3) was used to remove water molecules, cofactors and original ligands from the crystal structure [31]. The small molecule ligand structure was drawn with ChemDraw 22.0.0, then converted into a 3D conformation and subjected to energy minimization via Chem3D [32]. Molecular docking was performed using AutoDock Vina 1.2.0 [33], with the docking grid size set to 60 × 60 × 60 Å^3^ and a grid spacing of 0.0375 Å. Redocking of the native ligand was carried out under identical docking parameters, and the RMSD value between the redocked conformation and the original ligand conformation was calculated by PyMOL (v1.3) software. Meanwhile, the peptide LDEETGEFL was selected as the positive control and subjected to MD under the same computational conditions. The binding modes and interactions were analyzed in detail using Discovery Studio 2019 [34], with a focus on identifying non-covalent binding characteristics including hydrogen bonds and hydrophobic interactions.

## 3. Results

### 3.1. Peptide Identification

Two identification strategies (database searching + de novo sequencing) were applied. Database searching identified 189, 641 and 628 peptides from P1, P2 and P3, respectively; deduplication across three fractions yielded 1177 unique peptide sequences; de novo sequencing detected 558, 1209 and 1909 peptides in P1, P2 and P3, with 4696 unique peptides after cross-fraction deduplication.

**Figure 1:**
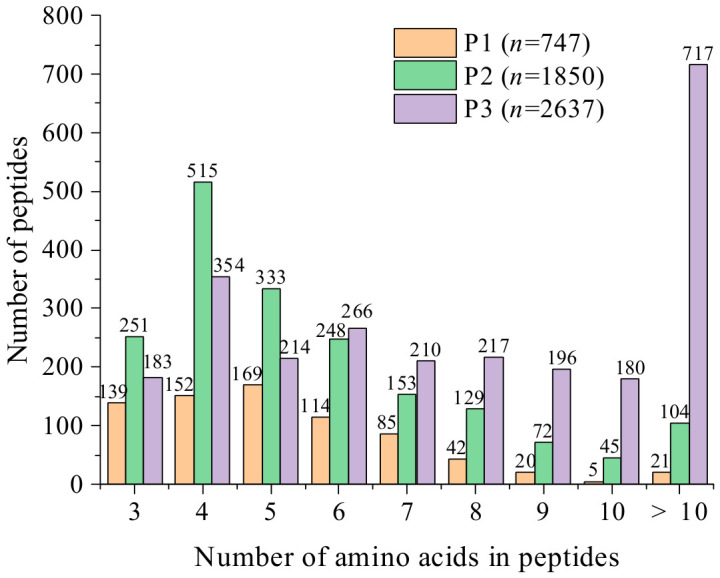
Comparison of number of amino acids in peptides from different sources.

Combined identification data were merged and deduplicated (Figure 1). P1 contained 747 peptides, predominantly short sequences of 3–6 amino acid residues; pentapeptides accounted for 22.62%, high-molecular-weight peptides (≥9 residues) occupied 3.5%, and no ultra-long peptides were detected. P2 yielded 1850 peptides with lengths ranging from 3 to 10 residues, dominated by 3–6-residue oligopeptides. P3 contained 2537 peptides, with long peptides (>10 residues) accounting for 28.3%. All detected peptides were categorized as small peptides (3–6 residues) and medium peptides (7–9 residues) based on sequence length.

### 3.2. Global Difference Analysis of Peptides

**Figure 2:**
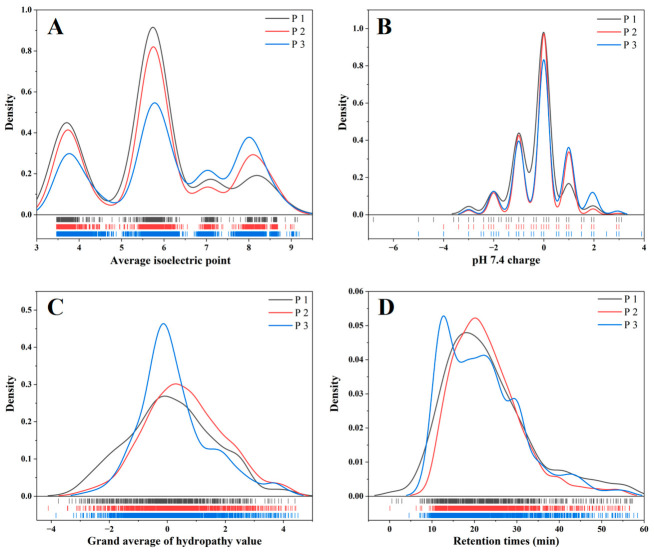
Comparison of axis distribution diagram from different peptide sources. (**A**) Average isoelectric point; (**B**) charge state in pH 7.4; (**C**) GRAVY value; (**D**) retention times.

Peptides from three fractions were compared across molecular weight (MW), pI, GRAVY value, net charge at pH 7.4 and chromatographic retention time (RT) (Figure 2). MW distribution varied significantly across groups: P1 peptides ranged from 301 to 1938 Da (mean = 1119 Da), with 74.16% of peptides at 300–700 Da and no peptides exceeding 2100 Da. P2 peptides spanned 301–3594 Da (mean = 1948 Da), containing a small proportion of peptides > 2100 Da. P3 peptides ranged from 315 to 3630 Da (mean = 1972 Da), with 35.55% at <700 Da and 44.89% at 700–1300 Da. pI ranges were 3.469–9.147 (P1), 3.466–9.066 (P2) and 3.469–9.187 (P3). At pH 7.4, net charge ranges were −6.8 to 3.1 (P1), −4 to 3 (P2) and −5 to 3.9 (P3). GRAVY values ranged from −3.75 to 4.4 (P1), −4.1 to 4.425 (P2) and −3.833 to 4.5 (P3); P2 exhibited the minimum GRAVY value (−4.1), and P3 exhibited the maximum value (4.5). For chromatographic RT, major elution peaks of P3 concentrated at 10–25 min, while P1 and P2 peaks were distributed primarily at 15–30 min.

### 3.3. Peptide Difference Analysis Based on Antioxidant Activity

Merged peptide datasets were screened via AnOxPePred 1.0 and PeptideRanker. Initial screening recovered 25 candidate peptides from P1, 58 from P2 and 41 from P3 (124 total candidates); cross-fraction deduplication retained 119 unique antioxidant candidates (Table S1). The optimal peptide AYPPPHP was derived from P2 with an AnOxPePred score of 0.65837, and six peptides exhibited scores > 0.6.

Sequence length and amino acid composition were compared across candidate antioxidant peptides. P1 candidates were dominated by tetrapeptides (43.48%), with hepta- and octapeptides each accounting for 13.04% and a mean MW of 641.11 Da. P2 candidates mainly consisted of 4–6-residue peptides (66.03%), with tetrapeptides as the most abundant subtype (30.19%) and a mean MW of 684.69 Da. P3 candidates were predominantly 5–8-residue and >10-residue peptides (71.43% total), with long peptides (>10 residues) occupying 11.43% and a mean MW of 807.38 Da.

Terminal hydrophobic and aromatic residues are key determinants of peptide bioactivity. Cumulative counts of hydrophobic and aromatic residues were 196 (P2), 145 (P3) and 76 (P1). Counts of peptides with single-terminal hydrophobic residues were 24 (P1), 54 (P2) and 35 (P3) (Table 1 and Table 2).

### 3.4. Molecular Docking Analysis

Molecular docking targeting Keap1-Nrf2 was performed to screen peptides regulating the core cellular antioxidant pathway via competitive binding. Among the 119 unique candidate peptides, 114 exhibited binding affinity < −5 kcal/mol (95.8%) (Table S2). The top 10 peptides with the strongest binding affinity are summarized in Table 3, with two- and three-dimensional docking interaction diagrams provided in Figure S1. The original ligand redocking obtained a binding affinity of −11.6 kcal/mol and an iRMSD value of 0.082 Å, and the positive control peptide LDEETGEFL yielded a binding affinity of −9.4 kcal/mol (Table 3).

**Figure 3:**
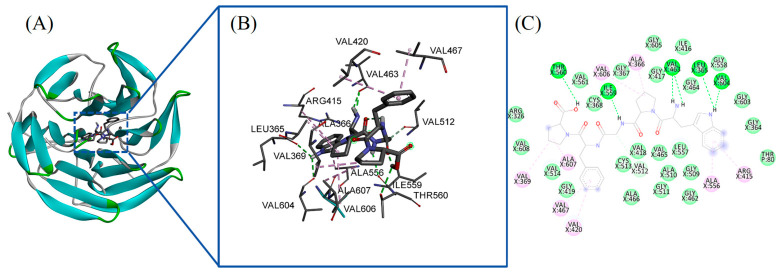
MD analysis of WPGFP with Keap1-Nrf2, including full docking poses (**A**), local binding views (**B**), and 2D interaction diagrams (**C**). In the local binding views, dashed lines of different colors represent key non-covalent interactions: pink dashed lines indicate alkyl and π-alkyl interactions, while purple dashed lines indicate π–σ interactions. In the 2D interaction diagrams, green dashed lines represent amino acid residues involved in van der Waals interactions with the ligand, pink dashed lines indicate residues involved in alkyl and π-alkyl interactions, and purple dashed lines indicate residues involved in π–σ interactions.

The binding affinities of the top 10 peptides ranged from −11.5 to −10.4 kcal/mol, with WPGFP showing the lowest binding affinity (−11.5 kcal/mol). As shown in Figure 3, WPGFP formed hydrogen bonds with five Keap1 residues (Thr560, Ile559), hydrophobic interactions with eight residues (Val369, Ala607), and van der Waals forces with 24 residues (Arg326, Val608). The multi-type non-covalent interaction network stabilized peptide–receptor binding.

## 4. Discussion

Traditional plant peptide identification relies solely on database searching, limited by incomplete plant genome annotation, species-specific peptide library deficiency and absent reference sequences for unannotated novel peptides. Previous single-database peptidomic profiling of PK only identified 37 peptides [6], while identical database searching recovered merely 286 endogenous peptides from its congener *P. odoratum* [35]. This study combined database searching and de novo sequencing to characterize the PK peptidome, identifying 4696 unique peptides—far exceeding yields of prior single-method analysis and establishing the largest PK peptide dataset to date [27]. Database searching alone detected far fewer peptides than de novo sequencing; the combined application compensates for the inherent limitations of individual analytical pipelines and significantly improves PK peptidome coverage and novel peptide discovery efficiency.

Differential pretreatment generated three PK peptide fractions with distinct abundance and length distributions. Fraction P1 exhibited the lowest total peptide yield, a narrow length distribution dominated by pentapeptides, and negligible high-MW peptides, consistent with native free peptide profiles of crude herbal extracts. Alkali extraction coupled with acid precipitation drastically increased peptide abundance in P2 (approximately 2.5-fold higher than P1), with uniform length distribution and enriched 7–10-residue peptides, ultra-long peptides, polar and basic amino acids. Alkalization disrupts plant cell walls to release matrix-bound peptides and low-MW proteins, while subsequent acid precipitation concentrates these components and improves MS detection of medium- and high-MW peptides [36]. Further enzymatic hydrolysis of alkali–acid-precipitated proteins yielded P3 with the maximum peptide count, enriched with both short and long peptides. Enzymatic degradation of macromolecular proteins generates peptides covering a broad MW range [37]. Most existing medicinal plant peptide studies adopt single extraction protocols, recovering only trace-free endogenous peptides and resulting in incomplete peptidome coverage without artificially processed exogenous peptides [37,38], restricting bioactive peptide discovery and resource exploitation potential. The three-step pretreatment system established in this study separately captures native free small peptides, matrix-bound medium/high-MW peptides and enzymatically derived novel exogenous peptides. Peptides from the three fractions exhibit complementary structural characteristics covering all molecular weights and binding states, constructing a comprehensive peptide library for antioxidant screening and providing material and theoretical foundations for subsequent functional verification and industrial utilization of PK antioxidant peptides.

The three PK peptide fractions shared overlapping physicochemical properties but diverged in MW, pI, GRAVY characteristics and chromatographic elution profiles. Molecular weight serves as the core structural feature that governs peptide charge, hydrophobicity, and active residue exposure. P1 and P2 were enriched in low-MW peptides (300–700 Da) with superior intestinal permeability and bioavailability [39], while P3 exhibited a reshaped MW profile with reduced small-peptide proportion and elevated medium-MW peptides; coexistence of short and medium peptides facilitates intestinal absorption and multi-target binding. All three fractions covered pI values spanning strongly acidic to weakly alkaline ranges with diverse charge properties. P1 displayed higher charge heterogeneity to adapt to variable microenvironments, while P2 and P3 showed concentrated charge distribution. The complementary charge subtypes among fractions alleviated peptide aggregation and inactivation under variable gastrointestinal pH conditions [40]. GRAVY values largely overlapped across groups, containing hydrophilic, hydrophobic and amphipathic peptides without extreme polar sequences. Balanced hydropathicity improves aqueous solubility and transmembrane transport, serving as a prerequisite for stable bioactivity [41]. Alkali–acid pretreatment enriched hydrophilic peptides in P2, while P3 contained peptides with the maximum GRAVY values, consistent with the shorter retention time of P3 major chromatographic peaks. Conventional single-preprocessing strategies generate peptide pools with homogeneous physicochemical properties, narrow MW distribution, limited charge subtypes and unbalanced hydrophilic–hydrophobic properties, impairing intestinal absorption, environmental stability and antioxidant functional diversity. The three complementary peptide fractions constructed in this study form a structurally diverse peptide library with strong environmental tolerance and balanced biological functions, enabling multi-pathway antioxidant activity via variable amino acid combinations and molecular architectures.

Two deep learning prediction tools (AnOxPePred 1.0 and PeptideRanker) were combined for systematic antioxidant peptide screening, realizing complementary functional evaluation and eliminating false-positive hits to reduce experimental workload compared with traditional activity screening pipelines. Amino acid sequences determine peptide bioactivity; terminal hydrophobic residues enhance antioxidant capacity, and abundant hydrophobic/aromatic residues generally strengthen radical-scavenging potency [41,42]. After deduplication, 119 candidate antioxidant peptides were identified, over 85% of which contained antioxidant-related hydrophobic and aromatic residues, verifying the reliability of screening thresholds. All three pretreatment groups yielded abundant bioactive candidates, confirming the necessity of each extraction strategy. Screened candidate peptides were predominantly short oligopeptides of 4–6 residues, consistent with findings from other *Polygonatum* species. Tetra- and pentapeptides purified from P. cyrtonema exert potent cytoprotection against H_2_O_2_-induced oxidative damage in HepG2 cells [43]; *P. odoratum* protein hydrolysates are dominated by 4–9-residue peptides, with 4–6-residue fragments exhibiting the strongest antioxidant activity [7]. Low-MW peptides from P. sibiricum show superior intestinal absorption and membrane permeability, with antioxidant activity significantly higher than long-chain fractions. Although di- and tripeptides occasionally display strong radical-scavenging capacity, they suffer from poor structural stability and short in vivo half-lives. Peptides of seven residues or longer readily form secondary structures that mask active amino acid sites, reducing radical contact and transmembrane permeability [44]. Tetra- to hexapeptides fully expose active residues and possess optimal biomembrane penetrability, enabling efficient molecular interactions and robust antioxidant responses—explaining their superior performance relative to long-chain peptides. These findings provide theoretical guidance for targeted preparation and processing optimization of high-activity short antioxidant peptides from PK.

Molecular docking was performed to further validate the antioxidant potential of PK peptides. Native ligand redocking was conducted to verify the reliability of the docking system, and the results showed a binding affinity of −11.6 kcal/mol and an iRMSD value of 0.082 Å, which fully met the standard criteria for credible molecular simulation and validated the accuracy and reliability of subsequent peptide docking results. Among 119 candidates, 114 peptides exhibited binding energies < −5 kcal/mol (95.8%), confirming stable binding between screened peptides and the Keap1 target. Binding energies ≤ −9.0 kcal/mol are defined as strong binding in molecular docking, indicating significant biological potential [33]. The positive control peptide LDEETGEFL presented a binding affinity of −9.4 kcal/mol in the present docking assay. Comparative analysis suggested that the top PK-derived peptides have strong binding affinity for Keap1, indicating they possess potential antioxidant activity. Among the top 10 high-affinity peptides, five originated from P1, three from P2 and two from P3, indicating endogenous PK peptides possess intrinsic antioxidant potential, and enzymatic hydrolysis further expands the pool of antioxidant peptide resources. Peptide–Keap1 interactions relied on hydrogen bonds, van der Waals forces and hydrophobic interactions, forming multi-layer non-covalent networks to stabilize binding. Amino acid composition analysis of the 10 peptides identified hydrophobic and aromatic residues (W, Y, F, P) as core binding motifs: these residues constituted 100% of sequences WVYP, WPF and VPPW, and exceeded 75% in the remaining seven peptides. Aromatic/hydrophobic side chains insert deeply into the hydrophobic Keap1 binding pocket and form stable non-covalent bonds with pocket key residues, serving as critical mediators of peptide–target-specific recognition. Peptides with the highest binding affinity were mostly 3–5-residue short sequences (WPF, WVYP, YFDW), while longer peptides (FSGGPPPPP, FPDWHSL) displayed weaker binding capacity. This phenomenon is attributed to the better spatial matching between short peptides and the Keap1 pocket, which reduces steric hindrance and optimizes binding conformation.

Published in vitro biochemical and cellular studies confirm that peptides with homologous core sequences to the candidates identified herein exert verified antioxidant activity. The tetrapeptide SGGY (core fragment of WSGGY) reduces intracellular ROS, activates the Nrf2 pathway and exerts neuroprotective effects against H_2_O_2_-induced oxidative stress in SH-SY5Y cells [45]. WVYP shares high sequence homology with hemp seed peptide WVYY, which regulates Nrf2 signaling to induce antioxidant responses in human keratinocytes [46]. The tripeptide WPF serves as a core active motif; long-chain peptides containing WPF exhibit strong hydroxyl radical-scavenging capacity [47]. WYGY is structurally analogous to egg yolk antioxidant peptide WYGPD, and sequences containing Trp (W) and Tyr (Y) show prominent in vitro radical-scavenging activity [48]. Current research on PK antioxidant components mainly focuses on polysaccharides and saponins. Low-MW PK polysaccharide PKP-1 alleviated free fatty acid-triggered oxidative stress in HepG2 cells by increasing SOD and CAT activities and GSH levels [49]. PK saponins trigger nuclear translocation of SKN1 and upregulate downstream antioxidant genes *gst4* and *sod3* [50]. To date, the antioxidant capacity and Keap1-regulating function of PK peptides remain uncharacterized. This study first predicts that PK peptides bind Keap1 to modulate the Nrf2 antioxidant pathway, filling the research gap regarding peptide-mediated Keap1 inhibition and confirming PK peptides as potential natural antioxidant agents.

Collectively, this study successfully screened high-affinity Keap1-Nrf2-targeted antioxidant peptides from three peptide sources of PK and clarified their amino acid composition and chain-length characteristics. This work not only speculated the molecular antioxidant mechanism of PK-derived peptides, but also provided a reliable material basis and theoretical foundation for the development of natural and potent Keap1-Nrf2 pathway activators and functional antioxidant peptides. This study has several limitations. First, all antioxidant activity evaluations solely relied on bioinformatic predictions and molecular docking simulations, without in vitro cellular antioxidant assays or in vivo animal model validation, thus lacking direct phenotypic evidence. Second, no synthesis of polypeptides was performed to obtain individual high-affinity active peptides, failing to quantitatively characterize the actual antioxidant capacity of each candidate peptide sequence. Third, only one pooled biological sample was used without independent biological replicates, so quantitative statistical analysis of inter-group peptide differences could not be implemented, and the reliability of comparative peptidomic profiling was limited. In follow-up research, we will prepare multiple independent PK batches as biological replicates and set parallel LC-MS technical replicates to support statistical testing of peptide abundance variation and reduce sampling uncertainty; meanwhile, we will synthesize core peptides with strong binding affinity and verify their antioxidant efficacy via cellular and animal experiments.

## 5. Conclusions

A hierarchical peptide processing system was established in this study. Three pretreatment strategies were integrated to process PK samples, and database searching coupled with de novo sequencing nano-LC-high-resolution MS was adopted to construct the largest peptidome dataset of PK to date. A total of 4696 unique peptides were identified, addressing the lack of high-coverage resources of species-specific peptides of PK in existing research. Two deep learning prediction models were applied to further screen 119 candidate antioxidant peptides, predominantly 3–5 amino acid-residue oligopeptides enriched in hydrophobic and aromatic amino acids. Molecular docking targeting the Keap1-Nrf2 axis revealed that 95.8% of candidate peptides exhibited strong binding affinity to Keap1 via hydrogen bonds, hydrophobic interactions and van der Waals forces. Short-chain peptides such as WPGFP displayed optimal pocket compatibility and binding stability. This study is the first to predict that endogenous and exogenous PK peptides exhibit activity against the Keap1-Nrf2 pathway and possess antioxidant potential, filling the research gap concerning the bioactivity of PK peptides. The integrated technical workflow developed herein provides a universal paradigm for mining bioactive peptides. The screened candidate short peptides serve as candidates for subsequent functional assays and facilitate the exploitation of PK resources.

## Figures and Tables

**Table 1:** Hydrophobic amino acid composition of P1, P2 and P3 peptides.

Amino Acids	P1 Peptides	P2 Peptides	P3 Peptides
Trp (Hydrophobic/Aromatic)	9	22	6
Phe (Hydrophobic/Aromatic)	11	30	36
Tyr (Hydrophobic/Aromatic)	13	36	24
Val (Hydrophobic)	0	9	6
Leu (Hydrophobic)	9	22	17
Ala (Hydrophobic)	2	6	4
Met (Hydrophobic)	4	2	2
Ile (Hydrophobic)	0	0	0
Pro (Hydrophobic)	28	69	50
Total	76	196	145

**Table 2:** Comparison of sequence of amino acids in peptides from P1, P2 and P3.

	P1 Peptides	P2 Peptides	P3 Peptides
Hydrophobic amino acids at both ends	18	34	16
Hydrophobic amino acids at single end	6	20	19
Total	24	54	35
Proportion	96.00%	93.10%	85.37%

**Table 3:** Molecular docking results of peptides interacting with Keap1-Nrf2 protein.

No.	Identification	Affinity (kcal/mol)	Hydrogen Bond	Carbon Hydrogen Bond	van der Waals	Hydrophobic Interaction	
						Pi-Sigma	Pi-Alkyl
1	WPGFP	−11.5	THR-560; ILE-559; VAL-463; LEU-365; VAL-604	VAL-512	ARG-326; VAL-608; VAL-514; GLY-419; VAL-561; CYS-368; CYS-513; ALA-466; VAL-418; GLY-367; GLY-417; GLY-605; VAL-465; ALA-510; GLY-511; ILE-416; GLY-464; LEU-557; GLY-509; GLY-462; GLY-558; GLY-603; GLY-364; THR-80		VAL-369; ALA-607; VAL-467; VAL-420; VAL-606; ALA-366; ALA-556; ARG-415
2	WVYP	−11.2	VAL-463; VAL-512; ILE-559; VAL-514; VAL-604; LEU-365	GLY-464; THR-560	ALA-510; ILE-416; GLY-509; LEU-557; GLY-511; GLY-417; GLY-558; VAL-418; GLY-367; GLY-419; VAL-467; CYS-513; VAL-420; VAL-561; VAL-608; GLY-603; GLY-605		ALA-556; CYS-368; ALA-607; VAL-369; ALA-366; VAL-606; ALA-466
3	WPF	−11.2	VAL-606; GLY-367; ARG-415; ALA-510	CYS-368; VAL-420; VAL-463	GLY-419; ALA-466; GLY-603; GLY-364; GLY-462; ALA-607; VAL-608; VAL-418; GLY-605; VAL-604; THR-560; ILE-559; GLY-558; GLY-464; VAL-512; GLY-511; GLY-417; VAL-465; LEU-557; ILE-416		VAL-467; ALA-556; ALA-366
4	YFDW	−11.1	VAL-606; LEU-557; VAL-604; VAL-463; VAL-512; THR-560; VAL-514; GLY-367	ALA-366; GLY-605; GLY-464	THR-80; GLY-509; GLY-364; GLY-603; ALA-510; GLY-462; ILE-416; LEU-365; GLY-417; GLY-558; CYS-513; GLY-511; VAL-465; VAL-561; ILE-559; VAL-418; ALA-607; VAL-369; GLY-419; ALA-466		ARG-415; ALA-556; CYS-368; VAL-420; VAL-467
5	FPQGY	−11.1	VAL-463; ILE-416; VAL-418; VAL-512; GLY-367; VAL-606	GLY-603; ALA-366; GLY-605; VAL-465	THR-80; GLY-364; LEU-365; GLY-462; GLY-511; ALA-510; GLY-509; GLY-558; GLY-464; VAL-608; ALA-466; GLY-419; VAL-420; VAL-467; VAL-514; THR-560; ILE-559; GLY-417; LEU-557		ALA-556; ARG-415; CYS-368; ALA-607; VAL-369; VAL-561; CYS-513
6	WYGY	−10.9	ILE-416; VAL-465; VAL-418; VAL-512; VAL-606	GLY-464; GLY-558; ILE-559; THR-560;	THR-80; GLY-462; GLY-509; GLY-603; VAL-463; ALA-510; GLY-511; LEU-557; LEU-365; GLY-367; VAL-604; GLY-605; VAL-420; VAL-608; VAL-369; CYS-368; ALA-466; VAL-514; VAL-467; GLY-419; CYS-513; GLY-417; GLY-364	ALA-607	ARG-415; ALA-556; ALA-366
7	WSGGY	−10.5	VAL-369; VAL-418; VAL-512; VAL-465; GLY-462; GLY-367; VAL-606; ILE-559; THR-560	CYS-513; GLY-464;	ARG-326; VAL-608; VAL-561; VAL-514; GLY-605; VAL-604; GLY-558; LEU-365; LEU-557; ILE-416; GLY-509; ALA-510; GLY-511; VAL-463; GLY-419; VAL-467; VAL-420; GLY-417; ALA-466	ALA-607	CYS-368; ALA-366
8	FSGGPPPPP	−10.5	ALA-510; LEU-557; ILE-416; GLY-367; ILE-559; THR-560	ARG-415; VAL-370; VAL-369; VAL-606; GLY-464	GLY-462; GLY-364; GLY-603; THR-80; GLY-371; GLY-372; GLY-423; VAL-420; ARG-326; GLY-605; GLY-509; ALA-366; LEU-365; GLY-511; VAL-463; GLY-558; GLY-417; VAL-465; VAL-512; VAL-418; VAL-561; VAL-608; GLY-419; CYS-368		ALA-556; CYS-513; ALA-607; VAL-514; VAL-467; ALA-466
9	VPPW	−10.5	VAL-606; GLY-367; VAL-463	THR-560	VAL-608; VAL-420; GLY-419; ALA-466; VAL-418; LEU-365; ILE-416; GLY-417; GLY-464; VAL-512; GLY-558; CYS-513; GLY-605; VAL-561; ILE-559		ALA-366; CYS-368; VAL-369; ALA-607; VAL-514
10	FPDWHSL	−10.4	VAL-512; GLY-367; VAL-606; ALA-510; ILE-416; VAL-604; LEU-365	LEU-557; GLY-417; THR-560; VAL-561	GLY-419; VAL-465; GLY-464; ALA-366; GLY-558; GLY-511; GLY-605; VAL-463; GLY-603; GLY-364; THR-80; GLY-509; GLY-462; CYS-368; VAL-418; VAL-608; ALA-466; VAL-514; GLY-423	ILE-559	VAL-420; VAL-369; VAL-467; ALA-556; ARG-415
11	AFFAQLQLDEETGEFL ^1^	−9.4	SER-555; ASN-382; ARG-380; ARG-483	TYR-334	GLY-419; VAL-608; GLY-417; GLY-511; GLY-558; ALA-466	TYR-572	TYR-525
12	LDEETGEFL ^2^	−11.6	SER-431; ASN-414; ARG-483; ASN-382; ASN-387; HIS-436		TYR-525; TYR-572; SER-602; GLY-603; SER-363; GLY-364; PHE-577; ILE-461; PHE-478; CYS-434; GLY-480; SER-555; ALA-556; PRO-384; SER-383		ALA-556; ALA-366; VAL-369; ALA-607

^1^ Co-crystallized 16-mer peptide of PDB 2FLU for redocking validation. ^2^ Positive control.

## Data Availability

All data generated or analyzed during this study are included in this published article [and its Supplementary Information Files].

## Supplementary Materials

The following supporting information can be downloaded at: https://www.mdpi.com/article/10.3390/cimb48070735/s1.

## Abbreviations

The following abbreviations are used in this manuscript:

PK	*Polygonatum kingianum*
MS	mass spectrometry
PSMs	peptide–spectrum matches
ALC	average local confidence
MW	molecular weight
pI	isoelectric point
GRAVY	grand average of hydropathicity
RT	retention time
MD	molecular docking
TFA	trifluoroacetic acid
DDA	data-dependent acquisition
NCE	normalized collision energy
