# Supplementary material for: Comprehensive Characterization and Antioxidant Function Prediction of Endogenous and Exogenous Peptides from Polygonatum kingianum
Source: Curr Issues Mol Biol. 2026 Jul 19;48(7):735. doi: 10.3390/cimb48070735 (PMC13408733; doi:10.3390/cimb48070735)
Supplement: Supplementary file 1 [file cimb-48-00735-s001.zip › cimb-4414303-supplementary.pdf]

Comprehensive Characterization and Antioxidant Function Prediction of Endogenous and Exogenous Peptides from *Polygonatum kingianum*

Jieyao Ma[1,†], Huiling Liu[1,†], Yalan Wu[1], Tingsheng Ma[1], Huaming Xiao[2,*],Wei Cai[1,*]

[1] School of Pharmaceutical Sciences, Hunan University of Medicine, Huaihua, Hunan, 418000, China; majieyao@hnmu.edu.cn (J.M.); 19372318251@163.com (H.L.); wuyalan5@163.com (Y.W.); matingsheng1111@163.com (T.M.); 20120941161@bucm.edu.cn (W.C.)

[2] School of Bioengineering and Health, Wuhan Textile University, Wuhan 430200, China; warmingshaw@whu.edu.cn

[*] Correspondence: 20120941161@bucm.edu.cn; Tel.:86-17375905466 (W.C.); warmingshaw@whu.edu.cn; Tel.: 86-13429858926 (H.X.)

[†] These authors contributed equally to this work.

Abstract

Background/Objectives: *Polygonatum kingianum* Collett & Hemsl. (PK) is a medicinal and edible plant with tonic effects. Its natural antioxidant peptides are valuable for functional food development, while their characteristics and mechanisms are unclear. This study aimed to identify PK antioxidant peptides and explore their antioxidant molecular mechanisms to support the utilization of PK active ingredients. **Methods:** Three peptide fractions (P1, P2, P3) were prepared from PK through defatting, alkaline acid precipitation and enzymatic hydrolysis. Nano-liquid chromatography-Q Exactive mass spectrometry was used for peptide identification. Bioinformatics analysis predicted peptide antioxidant activity, and molecular docking targeting the Keap1–Nrf2 pathway verified the binding ability of active peptides. **Results:** In total, 747, 1850 and 2537 peptides were identified from P1, P2 and P3, respectively, among which 119 were predicted to be antioxidant. Docking results showed ten peptides had strong Keap1 binding affinity. These active peptides were 3–5 residues short sequences enriched in hydrophobic and aromatic amino acids, which could stably bind key residues of the Keap1 pocket. **Conclusions:** This study elucidates the Keap1-Nrf2 pathway-based antioxidant mechanism of PK peptides. The screened short active peptides are excellent natural antioxidant candidates. The findings reveal PK's antioxidant mechanism and provide valuable resources for its industrial application and novel antioxidant peptide exploration.

Keywords: *Polygonatum kingianum*; peptides; Nano-liquid chromatography-high resolution mass spectrometry; bioinformatics analysis, molecular docking

Table S1. Molecular weight, FRS score and Peptide Ranker Value of candidate peptides from P1, P2 and P3 samples.

Sample No.	Peptide No.	Peptide sequence	Molecular Weight	FRS score	Peptide Ranker Value
P1	1	PFPFPLP	813.4425	0.52581	0.988034
P1	2	PFPFPLPL	926.5266	0.51327	0.987944
P1	3	FPFPLPL	829.4738	0.50097	0.986055
P1	4	GPLW	471.2481	0.51039	0.976889
P1	5	YFDW	671.2591	0.50919	0.969195
P1	6	FFCGGY	734.2734	0.55273	0.965588
P1	7	MYFG	558.2148	0.53527	0.95114
P1	8	WEGW	576.2332	0.54961	0.938658
P1	9	YGCC	444.1137	0.51601	0.93725
P1	10	WGPTPP	669.3122	0.55646	0.915953
P1	11	YGPP	432.2009	0.58636	0.886021
P1	12	WSGGY	584.2231	0.54272	0.883676
P1	13	FPQGY	610.2751	0.53388	0.882302
P1	14	GCYYMN	749.2513	0.58003	0.860695
P1	15	PHPP	462.2227	0.58375	0.826623
P1	16	ALPPY	559.3006	0.51924	0.778729
P1	17	WTGY	541.2172	0.55113351	0.771032
P1	18	HHMM	586.1992	0.5223	0.752086
P1	19	WGDHGKP	811.3613	0.5659	0.739863
P1	20	YYP	441.19	0.64273	0.724087
P1	21	APHP	462.2227	0.54656	0.720433
P1	22	HDGGGHLW	935.3885	0.5374	0.715019
P1	23	CCECGHLP	876.2928	0.51425	0.703157
P1	24	YGW	424.1747	0.59079	0.970785
P1	25	PFLPFP	716.3897	0.50501	0.983684
P2	1	WFP	490.2216	0.53168	0.997074
P2	2	WPGFP	660.2907	0.55574	0.992464
P2	3	PWCP	501.2046	0.55127	0.988949
P2	4	FPPFPLP	813.4425	0.52275	0.988242
P2	5	PFPFPLP	813.4425	0.52581	0.988034
P2	6	FYGGF	589.2536	0.57882	0.985165
P2	7	YSWPGLF	868.4119	0.57112	0.973497
P2	8	DHWWL	797.3497	0.59694	0.965638
P2	9	GFGY	484.1958	0.53015	0.952428
P2	10	FYFLH	725.3537	0.54824	0.950426
P2	11	HDPCW	656.2377	0.50677	0.945435
P2	12	FSGGPPPPP	851.4177	0.61547	0.940675
P2	13	WYGY	587.238	0.65336	0.936569
P2	14	VPPW	555.2693	0.54155	0.931491
P2	15	LWGGHLF	828.4282	0.59114	0.927747
P2	16	GAWPDLYL	933.4596	0.52345	0.926149
P2	17	GYAF	456.2009	0.5019	0.91147
P2	18	GFGGY	541.2172	0.53593	0.897977
P2	19	VVPGYYWW	1100.4967	0.65691	0.893136
P2	20	GFHY	564.2332	0.59369	0.886618

P2	21	FSYP	512.2271	0.53512	0.878003
P2	22	VWGP	457.2325	0.54388	0.875047
P2	23	GFEYVPF	899.4065	0.53736	0.869226
P2	24	VYYPF	687.3268	0.64143	0.865243
P2	25	YGSNPPHLF	1030.4872	0.56373	0.863322
P2	26	AYPPPHP	777.3809	0.66027	0.852783
P2	27	GYGGPP	546.2438	0.60418	0.846419
P2	28	PHEPPHLF	1030.4872	0.61352	0.845527
P2	29	PFAGGY	610.2751	0.52204	0.838173
P2	30	VLLPPW	723.4319	0.52374	0.836402
P2	31	YGPY	498.2114	0.6174	0.828168
P2	32	PHPP	462.2227	0.58375	0.826623
P2	33	YNGPPP	643.2966	0.57141	0.81446
P2	34	HLMYKF	837.4207	0.50247	0.81261
P2	35	HGPTF	557.2598	0.50427	0.80842
P2	36	DWH	456.1757	0.54332	0.806723
P2	37	WVYP	563.2744	0.58385	0.803226
P2	38	YGNSPPHLF	1030.4872	0.55633	0.802083
P2	39	KYLW	624.3271	0.50287	0.787249
P2	40	CPPDH	567.2111	0.50158	0.784381
P2	41	YGYP	498.2114	0.63344	0.78387
P2	42	SGNPPHL	720.3555	0.54367	0.776885
P2	43	WTGY	541.2172	0.55113	0.771032
P2	44	AFDYGNPPHL	1171.5298	0.60084	0.759751
P2	45	HHLF	552.2808	0.509	0.757393
P2	46	PYSDWH	803.3239	0.54063	0.747813
P2	47	GLVHPF	710.3751	0.54115	0.741521
P2	48	AYMY	588.2254	0.55169	0.735686
P2	49	DDWGYK	782.3235	0.53486	0.730962
P2	50	KPCHP	580.2791	0.52093	0.721286
P2	51	LDWH	569.2598	0.53988	0.715458
P2	52	ESYPPLG	761.3596	0.57188	0.714873
P2	53	HCPPEP	678.2795	0.51708	0.702563
P2	54	FPSHGKP	768.3918	0.53159	0.776059
P2	55	YLGP	448.2322	0.53348	0.724324
P2	56	YGL	351.1794	0.50793	0.724851
P2	57	FGY	385.1638	0.51315	0.96561
P2	58	FHW	488.2172	0.56163	0.990882
P3	1	WPF	464.206	0.53329	0.997303
P3	2	PFPFPLP	813.4425	0.52581	0.988034
P3	3	GEFPFP	734.3275	0.50643	0.959723
P3	4	FEGPF	637.2748	0.50274	0.945246
P3	5	GEFPFPRPP	1084.5342	0.54647	0.937122
P3	6	FPRPPH	749.3973	0.56274	0.900048
P3	7	DYLPYF	816.3694	0.57991	0.898012
P3	8	YDGW	539.2016	0.5294	0.896589
P3	9	FPDWHSL	900.413	0.52766	0.89384
P3	10	ALHPGYGFL	973.5021	0.61155	0.888241
P3	11	LPPYLP	698.4003	0.56063	0.886006

P3	12	GEFPFPRPPH	1221.593	0.53483	0.879841
P3	13	SFYYGKGL	933.4596	0.55991	0.864556
P3	14	GEFPFRPPPH	1221.593	0.52331	0.85372
P3	15	EPGEPFF	821.3596	0.50475	0.851215
P3	16	AFDWHSL	916.4079	0.51135	0.84073
P3	17	FGMAYGGCK	974.399	0.55682	0.839533
P3	18	GSSFYYGK	907.4076	0.55717	0.82722
P3	19	SFYYGK	763.3541	0.60388	0.821459
P3	20	FSYY	578.2377	0.56565	0.807797
P3	21	AFFHDY	798.3337	0.53388	0.805545
P3	22	YGNSPPHLF	1030.4872	0.55633	0.802083
P3	23	GYYP	498.2114	0.65837	0.79726
P3	24	NPPPPPVH	895.4552	0.55288	0.796489
P3	25	TYGLW	638.3064	0.51804	0.772017
P3	26	NPPPPVPH	895.4552	0.50945	0.771852
P3	27	HVPW	537.27	0.54183	0.7627
P3	28	EHLPFGL	811.4228	0.50063	0.75367
P3	29	FSYYGK	763.3541	0.61304	0.752422
P3	30	HDHGF	627.2401	0.54758	0.742053
P3	31	EGGFY	571.2278	0.51709	0.738413
P3	32	LMFPRPPH	993.5218	0.55668	0.735107
P3	33	VYPSF	611.2955	0.50489	0.725487
P3	34	KGGGHELLFVL	1168.6604	0.50302	0.721479
P3	35	QVGGYF	669.3122	0.52369	0.708247
P3	36	LLPW	527.3107	0.50885	0.944763
P3	37	LPYP	488.2635	0.58273	0.8183
P3	38	QGYF	513.2224	0.51587	0.921305
P3	39	TYPF	526.2427	0.52956	0.853096
P3	40	WHI	454.2328	0.54416	0.769297
P3	41	WHL	454.2328	0.55524	0.913034

Table S2. Binding affinities of 119 potential antioxidant peptides from molecular docking.

No.	Peptide sequence	Affinity (kcal/mol)	No.	Peptide sequence	Affinity (kcal/mol)	No.	Peptide sequence	Affinity (kcal/mol)
1	WPGFP	-11.5	41	VYYPF	-9.5	81	LLPW	-8.6
2	WVYP	-11.2	42	DWH	-9.5	82	LWGGHLF	-8.5
3	WPF	-11.2	43	LDWH	-9.5	83	GLVHPF	-8.5
4	YFDW	-11.1	44	QVGGYF	-9.5	84	DHWWL	-8.4
5	FPQGY	-11.1	45	YGPP	-9.4	85	FSYYGK	-8.4
6	WYGY	-10.9	46	HDPCW	-9.4	86	AYMY	-8.2
7	WSGGY	-10.5	47	GFGGY	-9.4	87	ESYPPLG	-8.2
8	FSGGPPPPP	-10.5	48	GFHY	-9.4	88	GEFPFP	-8.2
9	VPPW	-10.5	49	CPPDH	-9.4	89	YGL	-8.1
10	WGPTPP	-10.4	50	TYPF	-9.4	90	YGCC	-7.9
11	FPDWHSL	-10.4	51	YYP	-9.3	91	PHEPPHLF	-7.9
12	WEGW	-10.3	52	GYAF	-9.3	92	AYPPPHP	-7.8
13	HGPTF	-10.3	53	KYLW	-9.3	93	DDWGYK	-7.8

14	YGYP	-10.2	54	HCPPEP	-9.3	94	FPFPLPL	-7.7
15	WTGY	-10.2	55	FPRPPH	-9.3	95	PYSDWH	-7.7
16	FHW	-10.2	56	EGGFY	-9.3	96	PFPFPLP	-7.6
17	WFP	-10.1	57	WHI	-9.3	97	HHMM	-7.5
18	YDGW	-10.1	58	VWGP	-9.2	98	FPPFPLP	-7.5
19	FFCGGY	-9.9	59	LPPYLP	-9.2	99	DYLPYF	-7.4
20	FYGGF	-9.9	60	EHLPFGL	-9.2	100	NPPPPVPH	-7.4
21	GFGY	-9.9	61	FPSHGKP	-9.2	101	FGMAYGGCK	-7.0
22	HHLF	-9.9	62	WHL	-9.2	102	GSSFYYGK	-7.0
23	FEGPF	-9.9	63	PWCP	-9.1	103	GCYYMN	-6.8
24	YGW	-9.9	64	YSWPGLF	-9.1	104	YGNSPPHLF	-6.7
25	GYGGPP	-9.8	65	PHPP	-9.1	105	HLMYKF	-6.6
26	PFAGGY	-9.8	66	SGNPPHL	-9.0	106	ALHPGYGFL	-6.6
27	SFYYGK	-9.8	67	EPGEPFF	-9.0	107	CCECGHLP	-6.5
28	HDHGF	-9.8	68	YLGP	-9.0	108	GAWPDLYL	-6.5
29	VYPSF	-9.8	69	HDGGGHLW	-8.9	109	YGSNPPHLF	-6.2
30	FSYP	-9.7	70	GFEYVPF	-8.9	110	SFYYGKGL	-5.8
31	YGPY	-9.7	71	FSYY	-8.9	111	VVPGYYWW	-5.7
32	WGDHGKP	-9.6	72	GYYP	-8.9	112	AFDYGNPPHL	-5.6
33	AFFHDY	-9.6	73	QGYF	-8.9	113	PFPFPLPL	-5.5
34	TYGLW	-9.6	74	AFDWHSL	-8.8	114	NPPPPPVH	-5.4
35	HVPW	-9.6	75	MYFG	-8.7	115	GEFPFPRPP	-4.9
36	FGY	-9.6	76	VLLPPW	-8.7	116	LMFPRPPH	-4.2
37	LPYP	-9.6	77	PFLPFP	-8.7	117	KGGGHELLFVL	-2.8
38	GPLW	-9.5	78	FYFLH	-8.6	118	GEFPFRPPPH	-0.6
39	ALPPY	-9.5	79	YNGPPP	-8.6	119	GEFPFPRPPH	-0.3
40	APHP	-9.5	80	KPCHP	-8.6			

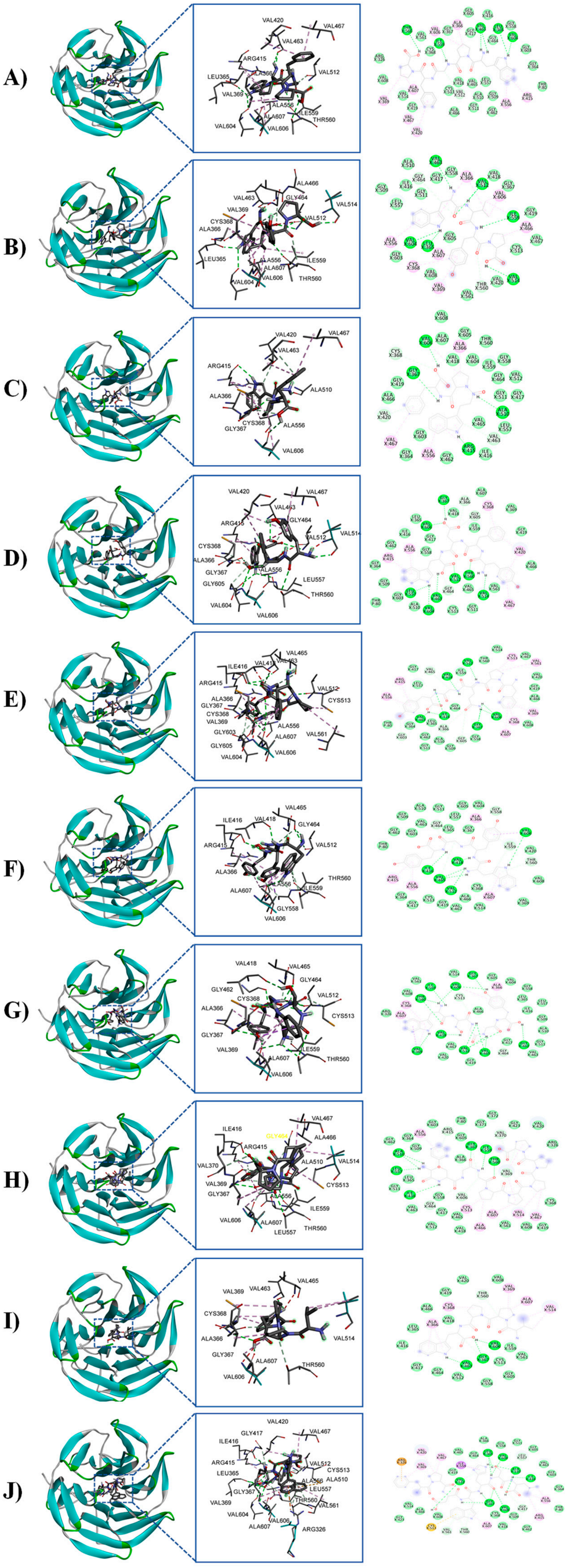
A)
VAL420
VAL467
VAL463
ARG415
VAL512
LEU365
ALA366
VAL369
ALA556
ALA607
ILE559
THR560
VAL604
VAL606
B)
ALA466
VAL463
GLY464
VAL514
VAL369
VAL512
CYS368
ALA366
LEU365
ALA556
ILE559
ALA607
VAL604
VAL606
THR560
C)
VAL420
VAL467
VAL463
ARG415
ALA510
ALA366
CYS368
GLY367
ALA556
VAL606
D)
VAL420
VAL467
VAL463
ARG415
GLY464
VAL514
CYS368
VAL512
ALA366
ALA556
GLY367
GLY605
LEU557
THR560
VAL604
VAL606
E)
VAL465
ILE416
VAL418
VAL463
ARG415
VAL512
ALA366
CYS513
GLY367
CYS368
VAL369
ALA556
GLY603
ALA607
VAL561
GLY605
VAL604
VAL606
F)
VAL465
ILE416
VAL418
GLY464
ARG415
VAL512
ALA366
THR560
ALA607
ALA556
ILE559
GLY558
VAL606
G)
VAL418
VAL465
GLY464
GLY462
VAL512
CYS368
ALA366
CYS513
GLY367
VAL369
ILE559
ALA607
THR560
VAL606
H)
VAL467
ILE416
GLY464
ALA466
ARG415
VAL514
VAL370
ALA510
VAL369
CYS513
GLY367
ALA556
ILE559
VAL606
ALA607
LEU557
THR560
I)
VAL369
VAL463
VAL465
CYS368
ALA366
VAL514
GLY367
ALA607
THR560
VAL606
J)
VAL420
GLY417
VAL467
ILE416
ARG415
VAL512
CYS513
LEU365
ALA510
ALA556
GLY367
LEU557
THR560
VAL369
VAL561
VAL604
VAL606
ALA607
ARG326

Figure S1. MD Analysis of Ten Compounds with Keap1-Nrf2, Including Full Docking Poses, Local Binding Views, and 2D Interaction Diagrams. Including WPGFP (A), WVYP (B), WPF (C), YFDW (D), FPQGY (E), WYGY (F), WSGGY (G), FSGGPPPPP (H), VPPW (I), FPDWHSL (J). In the local binding views, dashed lines of different colors represent key non-covalent interactions: pink dashed lines indicate alkyl and π-alkyl interactions, while purple dashed lines indicate π–σ interactions. In the 2D interaction diagrams, green dashed lines represent amino acid residues involved in van der Waals interactions with the ligand, pink dashed lines indicate residues involved in alkyl and π-alkyl interactions, and purple dashed lines indicate residues involved in π–σ interactions.
